# Analysis of illegitimate genomic integration mediated by zinc-finger nucleases: implications for specificity of targeted gene correction

**DOI:** 10.1186/1471-2199-11-35

**Published:** 2010-05-10

**Authors:** Petter A Olsen, Monika Gelazauskaite, Markus Randøl, Stefan Krauss

**Affiliations:** 1 Section for Cellular and Genetic Therapy, Institute of Microbiology, Oslo University Hospital, Rikshospitalet, Gausdadalleen 21, 0349 Oslo, Norway; 2 University of Oslo, 0027 Oslo, Norway

## Abstract

**Background:**

Formation of site specific genomic double strand breaks (DSBs), induced by the expression of a pair of engineered zinc-finger nucleases (ZFNs), dramatically increases the rates of homologous recombination (HR) between a specific genomic target and a donor plasmid. However, for the safe use of ZFN induced HR in practical applications, possible adverse effects of the technology such as cytotoxicity and genotoxicity need to be well understood. In this work, off-target activity of a pair of ZFNs has been examined by measuring the ratio between HR and illegitimate genomic integration in cells that are growing exponentially, and in cells that have been arrested in the G2/M phase.

**Results:**

A reporter cell line that contained consensus ZFN binding sites in an *enhanced green fluorescent protein *(*EGFP*) reporter gene was used to measure ratios between HR and non-homologous integration of a plasmid template. Both in human cells (HEK 293) containing the consensus ZFN binding sites and in cells lacking the ZFN binding sites, a 3.5 fold increase in the level of illegitimate integration was observed upon ZFN expression. Since the reporter gene containing the consensus ZFN target sites was found to be intact in cells where illegitimate integration had occurred, increased rates of illegitimate integration most likely resulted from the formation of off-target genomic DSBs. Additionally, in a fraction of the ZFN treated cells the co-occurrence of both specific HR and illegitimate integration was observed. As a mean to minimize unspecific effects, cell cycle manipulation of the target cells by induction of a transient G2/M cell cycle arrest was shown to stimulate the activity of HR while having little effect on the levels of illegitimate integration, thus resulting in a nearly eight fold increase in the ratio between the two processes.

**Conclusions:**

The demonstration that ZFN expression, in addition to stimulating specific gene targeting by HR, leads to increased rates of illegitimate integration emphasizes the importance of careful characterization of ZFN treated cells. In order to reduce off-target events, reversible cell cycle arrest of the target cells in the G2/M phase is an efficient way for increasing the ratio between specific HR and illegitimate integration.

## Background

Currently, gene targeting by homologous recombination (HR) is the standard method utilized for precise genome modification of mammalian cells. In this strategy the cellular DNA repair pathway, HR, mediates exchange of sequences between a given donor DNA sequence and a homologous genomic target sequence [1]. Although conventional gene targeting is very successful in some applications like in the creation of transgenic mice, the generally low frequencies of specific targeting events makes the use of the technique limited to situations where powerful selection and screening schemes can be applied [2,3]. Technologies for obtaining high frequencies of targeted sequence alteration in living cells have far reaching implications for the construction of transgenic cell lines and animals, both for the study of gene function and for establishment of disease models. Furthermore, direct modification of a target gene at its genomic loci at high frequencies offers an appealing strategy for gene therapy.

**Figure 1:**
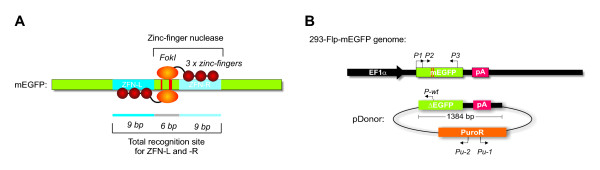
**Schematic representation of the model system**. (**A**) Features of the *mEGFP *coding sequence (green) with binding sites for ZFN-Left (L) and ZFN-Right (R) indicated (dark and light blue, respectively). The positions of the mutations rendering the *mEGFP *gene non-functional are shown in red. The ZFN-L and ZFN-R bound to their target sequences are indicated, each with their zinc-finger domains (3 × red circles) and the FokI nuclease domain (orange circle). The two 9 bp long recognition sites for ZFN-L (GGG GTA CGC) and ZFN-R (GAA GCA GCA) are separated by a 6 bp spacer region required for optimal positioning of the two FokI nuclease domains. Given that the FokI nuclease domain must dimerize in order for a DNA DSB to be formed, the total recognition sequence required for DSB formation is 18 bp, excluding the 6 bp spacer region. The 293-Flp-mEGFP cell line contained a single copy chromosomally integrated *mEGFP *gene. (**B**) Arrangement of the *mEGFP *gene and surrounding genetic elements in the 293-Flp-mEGFP cell line including the human Elongation Factor 1a promoter (EF1a, black), the *mEGFP *gene (green) with the point mutations (red) and the poly-adenylation signal (pA, red). The pDonor construct, provided as a substrate for HR, is shown as a circle with the regions homologous to the 293-Flp-mEGFP genomic sequence as indicated (*ΔEGFP *and pA, 1384 bp in total). The expression cassette for the *PuroR *gene is outlined in orange. Position and direction of the PCR primers used (P1, P2, P3, P-wt, Pu-1 and Pu-2) are indicated. With this model system, HR between the genomic *mEGFP *gene in the 293-Flp-mEGFP cells and the *ΔEGFP *gene in the pDonor construct could be measured by quantification of green fluorescent cells, while the rate of illegitimate integration could be quantified by appearance of puromycin resistant clones.

With the aim of developing methods for more efficient and specific gene targeting, various alternative strategies have been exploited including the use of modified donor DNA, recombinant enzymes and modified viral vectors [4-6]. Nevertheless, the most successful approach by far is based upon stimulating HR in the targeted cells through formation of site specific DNA double-strand breaks (DSBs). It has long been known that the formation of DSBs in a target gene increases the rates of HR by up to three orders of magnitude [7]. Lately, a strategy based on the capacity of zinc-finger nucleases (ZFNs) to introduce site specific DSBs has successfully been employed to substantially increase gene targeting rates [8-11]. ZFNs are engineered proteins composed of modular zinc-finger DNA binding domains coupled to the catalytic domain from the FokI restriction endonuclease that will induce site specific DSBs [12]. Each zinc-finger domain can specifically interact with three base pairs (bp) in the target DNA and due to the requirement of the FokI catalytic domain for dimerization, the composed recognition site for a pair of three-finger ZFNs will be 18 bp (Fig. 1A) [11,13]. Thus, by modifying the zinc-finger DNA binding domains, it is possible to generate ZFNs that can induce formation of specific DSBs in a broad range of DNA sequences. Targeted genome modification by ZFNs has successfully been achieved in cells form several species including *Caenorhabditis elegans *[14], *Drosophila *[15], zebra fish [16,17], plants [18,19], rats [20] and humans (including embryonic and induced pluripotent stem cells) [8,21,22].

For practical applications of ZFN induced HR it is essential that all possible adverse effects that ZFNs and donor plasmids might have on the target genome are well characterized. Formation of off-target genomic DSBs could cause unwanted effects on the targeted genome including, in the worst case, transformation due to genetic rearrangements [23-25]. In this work the relation between HR and non-homologous integration after ZFN treatment was investigated in a model system based on a mutated *enhanced green fluorescent protein *(*mEGFP*) gene in human embryonic kidney 293 cells. The donor plasmid, given as a template for HR repair of the DSB formed, also included an expression cassette for the *puromycin resistance *(*PuroR*) gene. Accordingly, by quantifying the number of puromycin resistant colonies formed with and without expression of the ZFNs, the level of illegitimate integration could be measured and used as an indication of the specificity of the ZFNs. In 293-Flp-mEGFP cells that contained the canonical ZFN binding sites and in wild type (wt) 293 cells devoid of the ZFN binding sites, the levels of illegitimate integration were increased 3.5 fold upon ZFN exposure. Analysis of the integrity of the *mEGFP *gene in puromycin resistant colonies showed that the *PuroR *expression cassette had not integrated at the position where the ZFNs were intended to create a DSB. Interestingly, co-occurrence of both specific HR and illegitimate integration could be observed in a fraction of ZFN treated cells. Importantly, transient cell cycle arrest in the G2/M phase during ZFNs exposure did not only boost rates of HR, but also increased the ratio of specific HR versus illegitimate integration by nearly eight fold.

## Results

### Stimulation of targeted gene correction by concurrent expression of ZFN-Left and ZFN-Right

To study the use of ZFNs in gene targeting, human 293 cells containing a single copy of a chromosomally integrated *mEGFP *gene were created and named 293-Flp-mEGFP. The *mEGFP *gene in the 293-Flp-mEGFP cell line does not encode for a functional EGFP protein due to the introduction of a premature stop codon together with a two bp deletion in the *EGFP *coding sequence (CDS) (Fig. 1A). The two ZFNs used in this study, ZFN-Left (L) and ZFN-Right (R) have been constructed by assembly of single zinc-finger modules as previously described [8]. The expressed ZFN proteins each consisted of a three-finger zinc finger DNA binding domain coupled to the non-specific catalytic domain from the FokI nuclease (Fig. 1A). Binding of the two ZFNs to their target sequences in the *mEGFP *gene would lead to the formation of a genomic DSB, a type of DNA damage that is known to stimulate HR when a homologous donor sequence is available as template for repair. The donor plasmid (pDonor) used contained a truncated wt *EGFP *gene (*ΔEGFP*) lacking the first seven bases of the *EGFP *CDS (Fig. 1B). In addition the pDonor construct incorporated an expression cassette for the *PuroR *gene that upon stable genomic integration would make the target cells resistant to selection with puromycin (Fig. 1B).

**Figure 2:**
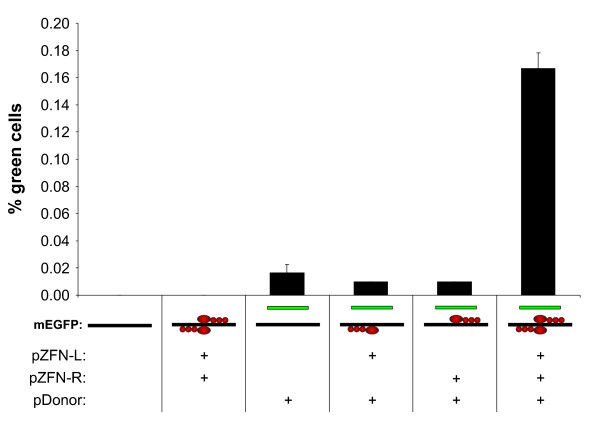
**Stimulation of targeted gene correction by ZFNs**. 293-Flp-mEGFP cells with a single genomic copy of the *mEGFP *target (black line) were transfected with the pDonor (green line) and ZFN constructs (four red circles) as indicated below the bars. Gene correction as a result of HR between the genomic *mEGFP *target and the *ΔEGFP *in the pDonor was measured 72 hours after transfection by flow cytometric quantification of green cells. For each flow cytometric analysis at least 100 000 cells were measured. Each bar represents mean ± SD from three parallels.

In non-transfected and in 293-Flp-mEGFP cells transfected with the ZFNs alone, no green cells could be detected (detection limit ~1 in 100 000) (Fig. 2). These results demonstrated that our cellular system was without leakage and with no spontaneous background fluorescence. When 293-Flp-mEGFP cells were transfected with the pDonor alone or with pDonor together with either ZFN-L or ZFN-R individually, 0.02% green cells were observed (Fig. 2). Given that control transfection of wt 293 cells with pDonor alone also produced 0.01% green cells (not shown) this level of fluorescent cells represented the background fluorescence produced from the pDonor construct. However, when both ZFNs were transfected together with the pDonor, the number of green cells increased by 9 fold (to 0.17%) compared to what was obtained with pDonor alone (Fig. 2). These results verified the previously reported stimulation of HR by ZFNs and also confirmed the dependence of binding of both ZFN-L and ZFN-R to their respective target sites in order to produce a specific DSB in the target *mEGFP *gene [9-11]. Although other authors have reported higher rates of ZFN mediated stimulation of targeted gene correction [8,26], our results confirmed the stimulation of HR by ZFNs and established the applicability of our model system to investigate ZFN mediated gene correction.

### Effects of ZFNs on illegitimate integration

**Figure 3:**
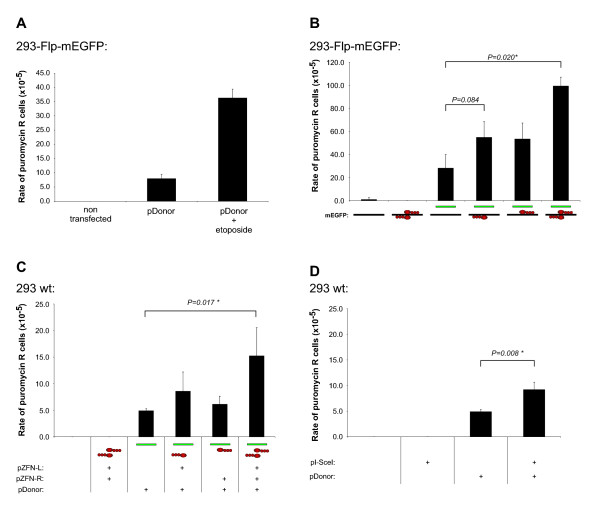
**Analysis of illegitimate genomic integration of the donor plasmid in ZFN treated cells**. (**A**) Effect of etoposide treatment on the levels of illegitimate integration of the *PuroR *expression cassette from the pDonor in the genome of 293-Flp-mEGFP cells. After transfection with pDonor, cells were either left untreated or treated with etoposide before selection with puromycin in order to quantify the levels of illegitimate integration. As a control for the selection conditions, non-transfected cells were also included. Each bar represents the mean rate of puromycin resistant (R) cells ± SD from three parallels. (**B**) Quantification of illegitimate integration in 293-Flp-mEGFP cells after transfection with pDonor along with pZFN-L and pZFN-R. Controls included non-transfected cells and cells transfected with pZFNs or pDonor individually as indicated below the bars. The rates of illegitimate integration were normalized according to variations in PE. Statistical P values (paired t-test) comparing the means of the indicated bars are shown and an asterisk marks significant difference (P < 0.05). (**C**) Analysis of illegitimate genomic integration of the pDonor in wt 293 cells that did not enclose the *mEGFP *target sequence in their genome. Cells were transfected with pDonor, pZFN-L and pZFN-R as indicated below the bars and analyzed as in B. (**D**) Analysis of illegitimate genomic integration of the pDonor in wt 293 cells after transfection with an I-SceI endonuclease expression construct (pI-SceI). Cells were transfected with pDonor together with 100 ng of pI-SceI. The frequency of illegitimate integration was quantified as in B.

With the aim of characterizing the specificity of the ZFNs, the levels of illegitimate integration of the pDonor was measured by quantifying the number of cells that acquired puromycin resistance after transfection of the pDonor along with the ZFNs. The rationale for this is that genomic DSBs tend to capture foreign DNA [27-29] and consequently, if the expression of ZFNs would induce the formation of off-target DSBs, increased levels of illegitimate integration of the pDonor would be expected. To verify that off-target genomic DSBs would lead to increased numbers of puromycin resistant colonies in our system, 293-Flp-mEGFP cells were transfected with pDonor and treated with etoposide, a topoisomerase II inhibitor known to induce the formation of DSBs [30]. As seen in Fig. 3A, etoposide treatment of 293-Flp-mEGFP cells transfected with pDonor resulted in a 4.6 fold increase in the number of puromycin resistant colonies. This verified that formation of genomic DSBs led to increased rates of illegitimate integration. Next, a potential general cytotoxicity of the ZFN-L and ZFN-R was quantified by measuring the plating efficiency (PE). Transfection of 293-Flp-mEGFP cells with ZFNs did not have a significant effect (P < 0.05) on the PE (not shown). Nevertheless, all further measurements of illegitimate recombination were normalized towards the PE obtained following the respective treatments.

Quantification of the number of puromycin resistant colonies formed after transfection of 293-Flp-mEGFP cells with various combinations of pDonor, ZFN-L and ZFN-R are shown in Fig. 3B. Transfection of pDonor alone produced a rate of 28 × 10^-5 ^puromycin resistant colonies (per treated cell). When either ZFN-L or ZFN-R was individually transfected with the pDonor, an increase in the frequencies of puromycin resistant colonies to 55 × 10^-5 ^was observed. Compared to the level of illegitimate integration obtained with the pDonor alone this increase was not statistically significant (P = 0.084). However, when 293-Flp-mEGFP cells were transfected with both ZFN-L and ZFN-R simultaneously together with pDonor, a significant increase (up to 99 × 10^-5^) of puromycin resistant colonies could be observed (P = 0.020). As a further control of the specificity of the ZFNs, their influence on illegitimate integration was also quantified in wt 293 cells that did not contain the *mEGFP *target gene incorporating the canonical ZFN binding sites. When wt 293 cells were transfected with either ZFN-L or ZFN-R individually along with the pDonor construct, no increase in illegitimate integration could be observed (Fig. 3C). However, in agreement with the results form the 293-Flp-mEGFP cell line, when both ZFN-L and ZFN-R were transfected together with the pDonor plasmid, a 3.6 fold increase in the number of puromycin resistant colonies was observed.

We next wanted to compare the observed ZFNs mediated increase of illegitimate integration with the effect of the I-SceI endonuclease. The yeast I-SceI endonuclease has an 18 bp recognition sequence that is rarely found in mammalian genomes and the endonuclease displays high specificity [7,31,32]. When the I-SceI expression construct were introduced into wt 293 cells together with the pDonor plasmid, a 1.8 fold increase in the frequency of illegitimate integration of the donor could be observed (Fig. 3D). As the protein expression levels of the ZFNs and I-SceI were not measured and as the cleavage kinetics of the ZFNs and the I-SceI might differ, the levels of illegitimate integration mediated by the ZFNs and I-SceI can not be compared directly. However, given that I-SceI is known to be very specific [7,31,32], the observed increase of illegitimate integration both by SceI (1.8 fold) and ZFNs (3.6 fold) when similar amounts of the expression constructs were used (100 ng pI-SceI versus 50 ng of each the two ZFNs) indicated that the specificity of the ZFNs and SceI are within a comparable range.

Thus, by measuring the levels of genomic integration of the donor plasmid it was established that expression of the here tested ZFNs produced a 3.5-3.6 fold increase of the levels of illegitimate integration. The relative increase of ZFN mediated illegitimate integration was found to be independent of the presence of canonical ZFNs binding sites in the genome of the targeted cells.

### Analysis of the genomic integration sites of the *PuroR *cassette in 293-Flp-mEGFP cells

**Figure 4:**
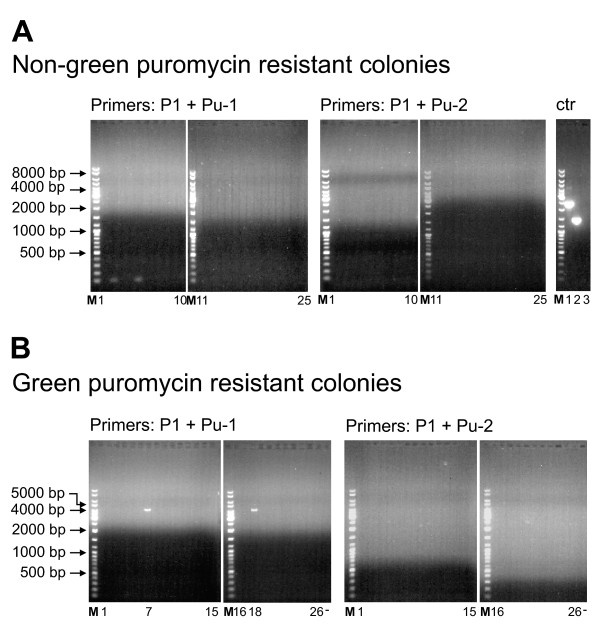
**Analysis of the *PuroR *genomic integration site in 293-Flp-mEGFP cells**. (**A**) The integrity of the *mEGFP *gene in non-green puromycin resistant colonies of 293-Flp-mEGFP cells was analyzed after transfection with pDonor together with the ZFNs. Genomic DNA isolated from 25 individual colonies was subjected to PCR using primer pairs as indicated above the panels. The genomic *mEGFP *specific primer (P1) was used together with the puromycin specific primers in opposite orientations (Pu-1 and Pu-2). Control PCR's (rightmost gel, ctr) using pEGFP-BABE-puro as template was performed with the primer pairs: P-amp + Pu-1 (lane 1), P1 + Pu-1 (lane 2), P1 + Pu-1 without template (lane 3) and PCR amplification produced the anticipated amplification products (2364 bp, 1429 bp and no product) verifying that primers worked under the given conditions. (**B**) PCR analysis of genomic DNA from 26 individual colonies displaying both green fluorescence (HR) and puromycin resistance (illegitimate recombination) after transfection with the pDonor and ZFNs. The genomic *mEGFP *specific primer (P1) was used together with the puromycin specific primers (Pu-1 and Pu-2) to investigate if the *ΔEGFP *and *PuroR *cassette had integrated in the genome of the 293-Flp-mEGFP cells as a single fragment or not. M represents the size marker and sizes of relevant bands are specified. Position of the PCR reactions from the individual colonies is indicated below the lanes.

To investigate whether the observed increase rates of illegitimate integration after ZFN exposure was due to an integration of the *PuroR *cassette into the genomic *mEGFP *gene, PCR analysis of the genomic DNA from 25 puromycin resistant colonies was performed. In two separate PCR analyses using the same forward primer (P1 in Fig. 1B), specific to the first bases in the genomic *mEGFP *gene, and primers specific for the *PuroR *gene either in forward or reverse orientation (Pu-1 or Pu-2 in Fig. 1B), no amplification products were obtained (Fig. 4A). In a positive control PCR using a plasmid template, the functionality of the primers and PCR conditions used were verified (Fig. 4A ctr). Accordingly, the failure to produce an amplification product from the genomic DNA was most likely due to the absence of the *PuroR *sequence in the *mEGFP *gene. To further test whether the genomic *mEGFP *in the puromycin resistant colonies was intact, PCR analysis of the puromycin resistant colonies with primers flanking the *mEGFP *gene (P2 and P3 in Fig 1B) was carried out. PCR analysis of the puromycin resistant colonies with primers P2 and P3 produced an amplification product of approximately 600 bp corresponding to the expected size of an uninterrupted *mEGFP *gene (610 bp) in all the 25 colonies (not shown). Taken together these results established that the increased rates of illegitimate integration following ZFN expression was not due to genomic integration of the *PuroR *cassette in the genomic *mEGFP *site where the ZFNs were designed to form a DSB. Given that that the average occurrence of the canonical 18 bp ZFN recognition sequence statistically is only one in the mammalian genome, the observed increased rates of illegitimate integration following ZFN expression was most likely due to formation off-target genomic DSBs by the ZFNs.

### Simultaneous occurrence of specific gene correction and illegitimate integration

Generally, the repair of genomic DSBs is carried out by either the HR or the non-homologous end joining (NHEJ) repair pathway [33]. However, the two pathways are not mutually exclusive, and crosstalk as well as co-operation between the two pathways has been demonstrated [34-36]. To determine whether both HR and illegitimate integration had occurred in individual cells transfected with ZFNs, an assay enabling the detection of both events in the same cells was established. After transfection of 293-Flp-mEGFP cells with the pair of ZFNs along with pDonor, green cells resulting from specific HR were isolated by fluorescent activated cell sorting (FACS). Subsequently, the green cells (where HR had occurred) and non-green cells (where HR had not occurred) were put under puromycin selection for quantification of the level of illegitimate genomic integration of the pDonor. When the frequency of illegitimate integration in the two populations was compared, similar levels of illegitimate integration were observed in both the green and the non-green populations of cells (not shown).

In the green fluorescent 293-Flp-mEGFP cells that also exhibited puromycin resistance, both the *ΔEGFP *and the *PuroR *regions from the pDonor construct must have been stably integrated into the genome of the target cells. Theoretically, the *ΔEGFP *and *PuroR *segments from the pDonor could either have been integrated jointly as a single fragment or they could have been integrated as multiple fragments. Given that the analyzed cells displayed green fluorescence due to HR between the genomic *mEGFP *gene and the *ΔEGFP *region in the pDonor, integration of the *ΔEGFP *and *PuroR *regions as a single fragment would require that the *PuroR *region to be positioned downstream (3') of the *mEGFP *gene (Fig. 1B). To study how the pDonor had integrated, PCR analysis of the *mEGFP *and 3' downstream regions was performed. When primer P1 (Fig. 1B), specific for the initial sequences of the genomic *mEGFP *target was used together with primers specific for the *PuroR *cassette in the donor (Pu-1 and Pu-2, Fig. 1B), PCR amplification products were obtained from 2 of the 26 clones analyzed (lane 7 and 18 in Fig. 4B). The size of the 2 amplification products were around 4500 bp which corresponds to the expected size (4389 bp) if the *ΔEGFP *and *PuroR *regions from the pDonor was integrated as a single fragment. The lack of PCR products in 24 of the analyzed clones indicated that the *PuroR *cassette has not integrated immediately downstream of the genomic *mEGFP *gene. As a control of the quality of the genomic DNA and as a confirmation of the FACS sorting, PCR analysis using primer P1 together with an allele specific primer (P-wt, Fig. 1B) only hybridizing to the wt *EGFP *was performed. An amplification product with the expected size (260 bp) was obtained in 24 of the 26 colonies analyzed (not shown).

Taken together, the analysis of the quality of the HR reaction established that in the majority (92%) of the clones that had undergone concurrent HR and illegitimate recombination, the *ΔEGFP *region and the *PuroR *region from the pDonor construct had been incorporated in the genome via multiple integration events. Only in 8% of the clones the *ΔEGFP *and *PuroR *regions had integrated as a single fragment "in frame" with the genomic *mEGFP*. Our observed genomic incorporation of both homologous and non-homologous sequences at a DSB in 8% of the clones is in accordance with Richardson *et al. *who demonstrated that a DSB repair event could be initiated by homologous invasion at one side and completed by NHEJ at the other end [36].

### Effects of transient G2/M cell cycle arrest on gene correction and illegitimate integration

**Figure 5:**
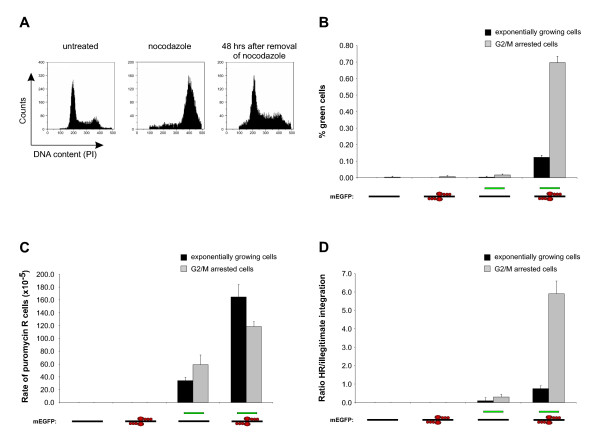
**Effects of G2/M arrest of the target cells on gene correction and illegitimate integration**. (**A**) Cell cycle profiles of exponentially growing, nocodazole arrested, and nocodazole arrested and subsequently released 293-Flp-mEGFP cells (left, middle and right panels, respectively). Histograms displaying the distribution of the DNA content of treated cells as measured by propidium iodide (PI) staining are shown. (**B**) Quantification of gene correction in exponentially growing (black bars) and G2/M arrested (gray bars) 293-Flp-mEGFP cells after transfection with pDonor, pZFN-L and pZFN-R as indicated. The amount of green cells was measured 72 hours after transfection by flow cytometry as in Fig. 2. (**C**) Quantification of illegitimate integration in exponentially growing (black bars) and G2/M arrested cells (grey bars). Cells were treated as in B and the level of illegitimate integration was quantified by measuring the number of puromycin resistant colonies formed as in Fig. 3B. The results presented are normalized for different PE in the untreated and G2/M arrested cells. (**D**) Presentation of the ratio between HR and illegitimate integration in exponentially growing (black bars) and G2/M arrested cells (grey bars). The ratio was calculated from the results in B and C.

Cell cycle status is an important factor in the decision whether a genomic DSB is repaired by HR or NHEJ. While NHEJ is active throughout the cell cycle, HR is mainly active in the late S and G2/M phases [37]. To examine the influence of cell cycle phase on the levels of both HR and illegitimate integration, 293-Flp-mEGFP cells were arrested in the G2/M cell cycle phase by the microtubule-depolymerizing drug nocodazole, that reversibly arrests cells in the G2/M phase [38]. As seen in Fig. 5A, incubation of cells with nocodazole for 24 hours resulted in a clear accumulation of cells in the G2/M cell cycle phase (middle panel). After removal and further incubation for 48 hours without nocodazole, cells reentered normal proliferation (right panel) and displayed a cell cycle profile equivalent to untreated cells (left panel). When the percentage of ZFN induced green cells was compared between exponentially growing and in G2/M arrested 293-Flp-mEGFP cells, a 5.8 fold increase of HR was observed in the G2/M arrested cell (from 0.12% to 0.70%) (Fig. 5B). The observed differences in HR was not due to differences in the transfection efficiencies as transfection with a wt *EGFP *expression plasmid (pEGFP-c1) displayed similar frequencies both in untreated and nocodazole treated cells (not shown). Increased rates of HR following nocodazole induced G2/M cell cycle arrest are in agreement with Urnov *et al*. who observed increased levels (4.8 fold) of ZFN mediated HR in cells reversely arrested in the G2/M phase using vinblastine [8].

When the rate of illegitimate integration was compared between exponentially growing and G2/M arrested cells, only minor variations was observed (Fig. 5C). Consequently, when the ratio of HR versus illegitimate integration was calculated, transfection of cells with pDonor and both ZFNs produced a nearly eight fold increased ratio in favor of HR in cells that had been arrested in the G2/M phase (Fig. 5D). Thus, reversible arrest of the target cells in the G2/M cell cycle phase can be an efficient way to increase the ratio between HR and illegitimate integration.

## Discussion

Stimulation of targeted genome modification by ZFNs has proven to be very successful [39]. Nevertheless, genotoxicity of ZFNs is still an important issue. Several reports have demonstrated substantial toxicity upon expression of ZFNs [10,26,40-45] while other publications claim relative minor levels of ZFN toxicity [8,15,46]. It is known that the formation of genomic DSBs induce cell cycle arrest and cell death [33], and a correlation between ZFN specificity and genotoxicity has been demonstrated [15,41]. In addition it has been shown that the presence of a genomic DSB triggers a multitude of DNA damage response signaling pathways and changes the activity of numerous central cellular proteins [47,48].

ZFN design, assembly and target sequences are important variables determining the specificity and potential genotoxic side effects of ZFNs [49-51]. Thus, two zinc-finger modules produce ZFNs with greater specificity compared to ZFNs assembled from single zinc-finger modules [52]. Publications using ZFNs assembled from two zinc-finger modules generally report low levels of cytotoxicity [8,18,22,46]. Also, generation of ZFNs by modular assembly of single-finger modules as provided by the Oligomerized Pool Engineering (OPEN) platform has generated efficient ZFNs with low levels of toxicity [19,53-56]. To study the genotoxicity of ZFNs various strategies have been employed. Quantification of cell survival [10,41,49] and apoptosis [26] offers a relatively crude measurement of potential side effects mediated by ZFNs, while quantification of genomic DSBs by detection of proteins normally recruited in DSB repair foci (e.g. γ-H2AX and p53 tumor suppressor binding protein 1) offers a more precise information to the mechanism that induce the toxicity [41,42,45,57]. We have previously used cell cycle analysis, quantification of γ-H2AX and direct detection of DSBs by a comet assay to assess ZFN mediated toxicity [58]. In the present work, the specificity of a pair of three-finger *EGFP *specific ZFNs has been investigated by measuring their influence on the levels of illegitimate integration of the given donor plasmid. We have shown that upon expression of the ZFNs in human 293 cells, the levels of illegitimate integration of the donor plasmid was increased by about 3.5 fold in cells containing the canonical ZFN binding sites and in cells devoid of the ZFN target sequences. In cells that contained the canonical ZFN binding sites, genomic analysis of the *mEGFP *target gene revealed that the donor DNA had not integrated at the position where the ZFNs where intended to form a DSB (Fig. 4A). Consequently, the observed increase in illegitimate integration upon expression of the ZFNs was most likely due to formation of off-target DSBs by the ZFNs. It has been beyond the scope of this work to characterize the precise mechanism leading to the formation of the off-target DSBs and therefore the integration sites of the *PuroR *cassette have not been mapped. However, it has been shown that formation of off-target DSBs by ZFNs can take place if ZFNs form homodimers, or if a pair of ZFNs binds to sites that are slightly different from the canonical 18 bp recognition sequence [59]. Furthermore, it is possible that off-target DSBs can form if only one ZFN binds to its 9 bp canonical recognition site while the second ZFN remains in solution, as observed with the native FokI restriction enzyme [60].

It is reaffirming for the technology, that we did not observe measurable cytotoxic effects on cells that had been transfected with the given ZFN constructs (no changes in PE). In addition, the levels of illegitimate integration obtained with the ZFNs, although present, were only moderately increased compared to the benchmark I-SceI endonuclease. Interestingly, Moehle *et al. *have recently shown that increasing doses of a pair of four-finger ZFNs did not lead to increased rates of illegitimate integration [46].

The main impediment in gene targeting approaches is the unfavorable ratio between high frequency of illegitimate integration and low frequency of specific HR [3]. By enabling the formation of genomic DSB in the target gene, ZFNs allow to significantly stimulate the rates of HR. However, as shown in this work, illegitimate integration still takes place and further means of increasing the rates of HR relative to illegitimate integration would be valuable. In the context of ZFN mediated gene correction, arresting cells in the G2/M phase has been shown to stimulate the rates of HR [8]. On the other hand, the influence of a G2/M arrest on the levels of ZFN mediated illegitimate integration has not been investigated. Our work showing that levels of illegitimate integration were almost unaffected by a reversible G2/M arrest of the target cells (Fig. 5C) is in agreement with publications showing that a G2/M cell cycle arrest did not influence on the activity of the NHEJ pathway [61]. Thus, the demonstration that reversible G2/M cell cycle arrests leads to a nearly eight fold increase in the ratio between specific HR and illegitimate integration (Fig. 5D) present an effective strategy to influence the activity of DNA repair pathways in order to achieve high levels specific ZFN mediated gene correction along with low levels of illegitimate integration.

By enabling the formation of site specific genomic DSBs over a range of genomic targets, the development of ZFNs has brought the field of targeted genome modification a substantial step forward. Next generation ZFNs with further improved properties [62] as exemplified by creation of ZFNs active only as heterodimers [42,45], along with generation of ZFNs with increased numbers of zinc-finger DNA binding modules [42,46,63,64], corroborate that ZFNs have a realistic potential for evolving into a valuable tool for functional genomics [20], biotechnology [18] and human gene therapy [57].

## Conclusions

The demonstration that ZFNs in addition to stimulate specific gene targeting by HR also can produce increased rates of illegitimate integration emphasizes the importance of cautious characterization of cells that have been treated with ZFNs. Reversible cell cycle arrest of the ZFN targeted cells in the G2/M phase is a simple and efficient way of positively altering the ratio between specific HR and illegitimate integration.

## Methods

### Plasmids

The ZFN target plasmid pcDNA4/TO/mutEGFP, and the expression plasmids for ZFN-L and ZFN-R (driven by CMV promoter) were generously provided by Samgamo BioSciences, Inc. (Richmond, California) and have been described elsewhere [8]. The *mutEGFP *(*mEGFP*) gene codes for a non-functional EGFP protein due to the presence of a premature stop codon (GAC to TAA) at position 229-231 and a two bp deletion (AT to --) at position 235-236 in the *EGFP *CDS. The pFRT/mEGFP construct used for making the 293-Flp-mEGFP cell line was made by cloning the *mutEGFP *sequence into pEF5/FRT/V5-D-TOPO (Invitrogen, Carlsbad, California). pFRT/EGFP (expressing wt EGFP) was made by cloning the wt *EGFP *CDS from pEGFP-c1 (Clontech Mountain View, CA) into the same construct. The pDonor construct incorporated a truncated *EGFP *sequence (promoterless) lacking the first seven bases (*ΔEGFP*) together with a *PuroR *expression cassette that had been cloned into pBluescript-SK (Stratagene, Cedar Creek, Texas) (Fig. 1B). The pEGFP-BABE-puro construct used in the control PCR in Fig. 4A was made by cloning the *EGFP *CDS from pEGFP-c1 into the pBABE-puro [65] construct. The expression plasmid for the I-SceI nuclease (driven by CMV promoter) was generously provided by Dr. Jasin [66]

### Cell lines

The wild type human embryonic kidney cell line (HEK 293, CRL-1572), was purchased from ATCC (ATCC-LGC, Boras, Sweden). The 293-Flp-mEGFP cell line, containing a single copy genomic integration of the mutated *EGFP *(*mEGFP*) gene, was generated by using Invitrogen's Flp-In system according to their instructions. Briefly, the stable 293-Flp-In cell line (Invitrogen, Carlsbad, California) that contains a single copy genomic integration of the Flp recombination target (FRT) site was transfected with the expression construct for the Flp recombinase (pOG44) together with the plasmid containing the *mEGFP *gene and a FRT site (pFRT/mEGFP). As a result of Flp recombinase mediated recombination, the *mEGFP *gene will be integrated in the genomic FRT site of the 293-Flp-In cells where its expression is driven by the human Elongation Factor 1α (EF1α) promoter (Fig. 1B). All cell lines were grown in DMEM containing 10% FBS and 1% penicillin-streptomycin at 37°C in a humidified atmosphere containing 5% CO_2_.

### Analysis of ZFN mediated genomic gene correction and illegitimate integration in 293-Flp-mEGFP cells

Cells were transfected in 12 well plates with pDonor (0.5 μg), pZFN-L and pZFN-R (0.05 μg each) as indicated in the figures. The total amount of DNA in each well was adjusted to be 0.6 μg by addition of the pBlueskript vector (Stratagene, Cedar Creek, Texas). Transfection was done using Lipofectamine (LF) 2000 (Invitrogen) according to the manufactures instructions (2.0 μl LF2000 pr. well, kept on the cells overnight). Two days after transfection, cells were plated out both in new 12 well plates for analysis of HR and in 60 mm dishes (600 000 cells/dish) for analysis of illegitimate integration. For analysis of HR, the amount of green cells was quantified (three days after transfection) on a Partec PAS flow cytometer (Partec GmbH, Münster, Germany). Cell sorting was performed with the FACSaria cell-sorting system (BD Biosciences, Franklin Lakes, New Jersey). For analysis of illegitimate integration of the pDonor plasmid, cells were grown in the presence of 1.0 μg/ml puromycin (Sigma-Aldrich, Inc., St. Louis, Missouri). Following 10 days of growth in selective medium, puromycin resistant colonies were visualized by methylene blue staining (0.2% in methanol, 5 min on the cells) and quantified using the GeneTools image analysis software (Syngene, Cambridge, UK).

### Etoposide treatment of cells

One day after transfection of 293-Flp-mEGFP cells with pDonor, cells were incubated overnight with growth medium containing 0.1 μM etoposide (Sigma-Aldrich). Three days after transfection the cells were plated in 60 mm dishes for puromycin selection and for measurement of the PE as described below. Etoposide treatment of the cells did not influence the PE (not shown).

### Quantification of cytotoxicity by measurement of plating efficiency (PE)

PE was determined by plating 20 cells/cm^2 ^in complete growth medium in 60 mm dishes. Following 14 days of expansion, the number of colonies formed was counted and the percentage of colony forming cells was calculated.

### PCR analysis of genomic integration sites

Following transfection of 293-Flp-mEGFP cells with pDonor together with ZFNs, analysis of the integrity of the *mEGFP *gene in non-green puromycin resistant 293-Flp-mEGFP cells was done by PCR analysis on their genomic DNA (Fig. 4A). Three days after transfection, non-green cells were isolated by FACS, replated and subjected to puromycin selection as described. Genomic DNA from the expanded colonies was isolated (Illustra blood genomicPrep kit; GE Healthcare, Chalfont St. Giles, UK) and used as template (0.5 μg) for PCR amplifications using the primers as indicated. Analysis of the quality of the HR reaction in cells displaying both green fluorescence and puromycin resistance (Fig. 4B) was performed on genomic DNA from 293-Flp-mEGFP cells that had been transfected with pDonor along with the ZFNs. The sequences of the primers used were the following (5' to 3'): P1: ata tcg gtc gcc acc atg gtg a; P2: ccc atc ctg gtc gag ctg ga; P3: cgc gct tct cgt tgg ggt ct; Pu-1: gcc cga cgc gcg tga gga aga gtt ctt; Pu-2: cgc gca tgg ccg agt tga g; P-amp: cca ctt ctg cgc tcg gcc ctt c; P-wt (gtc gtg ctg ctt cat gtg gtc). PCR amplifications were performed using the Phusion DNA high fidelity DNA polymerase (Finnzymes, Espoo, Finland) using the following cycling parameters: 98°C for 2 min, 30 cycles of 98°C for 15 seconds (sec), 72°C for X min/sec and final elongation at 72°C for 7 min. The elongation time (X) varied according to the primer pairs used. For amplification using the P1 together with Pu-1 or Pu-2 X was 4 min. For PCR with primer P2 and P3, X was 30 sec and in the control PCR using P1 + Pu-1 and P-amp + Pu-2, X was 2 min.

### Transient G2/M arrest and cell cycle analysis

293-Flp-mEGFP cells were arrested in the G2/M phase by incubation with nocodazole (Sigma-Aldrich). Nocodazole (0.1 μg/ml) was applied to the cells together with the transfection mixture and was kept on the cells for 24 hours before the cells were allowed to re-enter cell cycling. Cell cycle analysis was done on fixed cells (70% EtOH) stained with propidium iodide (PI) (20 μg/ml PI, 200 μg/ml RNaseA, 0.1% Triton-X100). Cells were replated for quantification of illegitimate integration and PE two days after transfection as described. The PE for exponentially growing cells was 33% while for G2/M arrested cells the PE was 5%.

## List of abbreviations

bp: base pairs; CDS: coding sequence; CMV: cytomegalovirus; DSBs: double-strand breaks; EF1α: elongation factor 1α; EGFP: enhanced green fluorescent protein; FACS: fluorescent activated cell sorting; FRT: Flp recombination target; HR: homologous recombination; LF: Lipofectamine; NHEJ: non-homologous end joining; OPEN: Oligomerized Pool Engineering; PE: plating efficiency; PI: propidium iodide; *PuroR*: puromycin resistance gene; wt: wild type; ZFNs: zinc-finger nucleases.

## Authors' contributions

PAO and SK designed the study and experiments. PAO carried out the majority of the experiments with the assistance of MR and MG. PAO was the primary author of the manuscript. SK conceived of the study and participated in its design, coordination and contributed to the writing of the manuscript. All authors approved the final manuscript.
